# A simulation study comparing supertree and combined analysis methods using SMIDGen

**DOI:** 10.1186/1748-7188-5-8

**Published:** 2010-01-04

**Authors:** M Shel Swenson, François Barbançon, Tandy Warnow, C Randal Linder

**Affiliations:** 1 Department of Computer Sciences, The University of Texas at Austin, Austin TX, USA; 2 Microsoft, Redmond WA, USA; 3 Section of Integrative Biology, The University of Texas at Austin, Austin TX, USA

## Abstract

**Background:**

Supertree methods comprise one approach to reconstructing large molecular phylogenies given multi-marker datasets: trees are estimated on each marker and then combined into a tree (the "supertree") on the entire set of taxa. Supertrees can be constructed using various algorithmic techniques, with the most common being matrix representation with parsimony (MRP). When the data allow, the competing approach is a combined analysis (also known as a "supermatrix" or "total evidence" approach) whereby the different sequence data matrices for each of the different subsets of taxa are concatenated into a single supermatrix, and a tree is estimated on that supermatrix.

**Results:**

In this paper, we describe an extensive simulation study we performed comparing two supertree methods, MRP and weighted MRP, to combined analysis methods on large model trees. A key contribution of this study is our novel simulation methodology (Super-Method Input Data Generator, or *SMIDGen*) that better reflects biological processes and the practices of systematists than earlier simulations. We show that combined analysis based upon maximum likelihood outperforms MRP and weighted MRP, giving especially big improvements when the largest subtree does not contain most of the taxa.

**Conclusions:**

This study demonstrates that MRP and weighted MRP produce distinctly less accurate trees than combined analyses for a given base method (maximum parsimony or maximum likelihood). Since there are situations in which combined analyses are not feasible, there is a clear need for better supertree methods. The source tree and combined datasets used in this study can be used to test other supertree and combined analysis methods.

## Background

Supertree methods-methods that, given a set of trees with overlapping sets of taxa, return a tree on the combined taxon set-offer one approach to estimating phylogenies from multi-marker datasets. Supertree estimation methods are of considerable interest in the systematics community, and several large phylogenies have been published using these methods [1].

Matrix representation with parsimony (MRP) [2,3] is currently the most widely used supertree method. It operates by encoding the set of source trees as a matrix of partial binary characters, one character for each branch of each source tree, and then analyzing that matrix using a parsimony heuristic. Weighted MRP [4] is a variant of MRP in which the partial binary characters are weighted, and this weighted matrix representation is then analyzed using weighted parsimony. These character weights are obtained from the source tree analyses, using either bootstrap support or posterior probabilities to assign weights to the branches of the source tree.

Several studies (mostly based upon simulation) have evaluated the performance of different supertree methods in terms of the topological accuracy of the resultant phylogenies and have investigated how different properties of the input-in particular, the percentage of missing data-and the method of phylogenetic analysis impact final phylogenetic accuracy [5-13]. Most of the supertree methods require that the input trees be rooted, a property that is not true of all trees in systematic studies, and potentially problematic because accurate rooting is itself a nontrivial issue. Of the supertree methods that do not require rooted input trees, MRP and weighted MRP are the most promising of the current supertree methods.

The main alternative to supertree methods are combined analysis methods, also known as supermatrix or total evidence approaches; these methods concatenate the alignments on each marker to produce a large "combined dataset", which is then analyzed using a phylogenetic estimation method (e.g., maximum likelihood or maximum parsimony). Sometimes a combined analysis is not always possible or advisable, e.g., when only source trees are available or when the source trees are derived from data types that cannot be used as input to a combined analysis [14-16]. These cases for supertree construction are not the subject of this paper.

Little is known about the relative accuracy of supertree and combined analysis approaches to multi-marker phylogenetics. Two studies have evaluated the relative performance of supertree methods and combined analysis methods: Bininda-Emonds and Sanderson [5], who evaluated combined analyses based upon maximum parsimony (MP), and Criscuolo et al. [13], who evaluated combined analyses based upon maximum likelihood (ML). Both found that combined analysis provided a somewhat more topologically accurate reconstruction than MRP. In addition, Bininda-Emonds and Sanderson found weighted MRP provided a slight improvement in tree accuracy over combined analysis when MP was used for both the source trees and the combined analysis. However, the experimental methodology of these earlier studies included elements that were neither biologically accurate nor reflective of systematic practice, so their conclusions regarding the relative performance of supertree methods and combined analysis need to be revisited. In particular, both studies examined datasets with small numbers of taxa (while supertree analyses are used primarily for very large numbers of taxa), and for their source trees, they chose taxa at random from their model trees (while most source tree datasets are generally based upon clades - sets of taxa densely sampled within one subtree of the tree). Given these methodological issues, the relative topological accuracy of supertree methods and combined analysis should still be considered open.

This paper introduces a novel simulation methodology, SMIDGen, which better reflects both biological processes and systematic practice than previous simulation techniques. We used SMIDGen to compare MRP, weighted MRP, and combined analysis on datasets with up to 1000 sequences. Under the conditions of our study, combined analysis using maximum likelihood consistently outperformed all other methods with respect to topological accuracy, suggesting that with more realistic simulations, MRP and weighted MRP supertree methods do not provide an acceptable alternative to combined analysis based upon maximum likelihood.

## Methods

### Experimental design

Estimating a species phylogeny from a dataset having multiple markers requires either a combined analysis or use of a supertree method. The availability of sequence data for each marker depends upon a number of factors, including biological processes (e.g., the novel gain of a gene within the evolutionary history and its loss in some lineages, and whether the marker evolves at the right rate for the taxa being reconstructed), and technical and practical issues (e.g., difficulties obtaining tissue samples for some taxa, difficulty successfully producing the sequence from some taxa, and the level of interest in a particular group). The consequence of these issues is that the pattern of missing data in both the supertree and combined analyses is not random.

Second, the source trees for a supertree method are produced by analyses of datasets selected by systematists, typically with the intent of estimating the phylogeny for a lower level taxonomic group (genera, families, and sometimes orders). We refer to these as "clade-based" studies. Clade-based studies usually have dense taxonomic sampling within the desired group (the ingroup), which tends to improve the ingroup's phylogenetic accuracy. For the taxa used as outgroups, sampling is almost always less dense. In addition to clade-based studies, systematists also produce "scaffold" phylogenies for higher level taxonomic groups, e.g., angiosperms [17] and metazoa [18,19]. Scaffold phylogenies sample taxa widely distributed across the group to provide a broad-scale sense of the relationships of the lower-level groups contained within the higher level group. In the context of a supertree analysis, scaffold phylogenies can provide the necessary "glue" for connecting phylogenies. In the absence of scaffold phylogenies, often the only overlapping taxa between source trees will be the small number of taxa used as outgroups for the clades of interest. Thus, supertree efforts will often consist of a large number of clade-based phylogenies that are densely sampled within their clades of interest and one or a small number of broadly distributed scaffold phylogenies based upon markers that evolve slowly (see Table 1).

**Table 1:** Selected empirical supertree studies

Group (Reference)	Method	Num. taxa	Num. source trees	Num. taxa in scaffold
Primates [34]	Hierarchical MRP	203	112*	203 (100%) (taxonomy)

Carnivora [35]	Hierarchical MRP	271	177*	not given

Hologalegina [36]	MRP (with topological constraints)	571	22	52 (9.1%)

Pinus [37]	MRP	95	14	47 (49.5%)

Bacteria [38]	wMRP	37	130-196	37 (100%)

Mammalia [39]	MRP (large:small source trees weighted 4:1)	90	430	37 (41.1%)

Procellariiformes [40]	MRP	122	7	90 (73.7%)

Chiroptera [41]	Hierarchical MRP	916	105	not given

Poaceae [42]	1) wMRP (normal, purvis, and irreversible)	403	55	not given

	2) wMRP (c.a. source trees)	61	8	61 (100%)

Lagomorpha [43]	MRP (robust:nonrobust source trees weighted 2.81:1)	80	146	not given

Lipotyphla [44]	(w)MRP (most source trees were MRP supertrees)	184	147 (7 final)	scaffold is a supertree of 6 taxa

Angiosperms [45]	wMRP	379	46	323 (85.2%), 224 (59.1%)

Marsupialia [46]	MRP (source trees with identical taxon sets were combined using supertree methods)	267	158	267 (100%) (taxonomy)

Cetartiodactyla [47]	MRP	290	201	290 (100%) (taxonomy)

Eutheria [48]	MRP (some source trees were MRP supertrees)	113	725 (109 supertrees)	115 (100%)

Carcharhiniformes: Sphyrnidae [49]	MRP (non-weighted, weighted, purvis, and irreversible)	8	5	8 (100%)

Mammalia [50]	MRP (combined previously published supertrees and some of their own.)	4510	>2500 (31 supertrees)	not given

Selected empirical supertree studies. For each supertree study we give the supertree method(s) used, the number of taxa in the final supertree, the number of source trees, and the number of taxa in the scaffold dataset. If we could not determine which source trees served as scaffold trees, we instead give the number of taxa in the largest source tree. * Indicates the number of publications from which source trees were drawn, not the actual number of source trees.

The simulation methodology we developed to reflect these biological and practical realities (SMIDGen) has six basic steps. In many cases we follow standard protocols, but with a few significant changes to increase simulation realism. The changes are indicated below.

#### Step 1: Generate model trees

We followed standard methodology here, generating trees under a pure birth process, and deviating these from ultrametricity (the molecular clock hypothesis). Unlike previous studies, we focused on large trees with 100 to 1000 taxa. We generated 30 replicates for each model tree size. However, we report the results for only 10 replicates for the 1000-taxon datasets due to the very long running times for the ML analyses of these datasets.

#### Step 2: Evolve gene sequences down the model tree

We modified the standard methodology for this step. We first determined the subtree within the model tree for which each gene would be present, using a gene "birth-death" process; this produced missing data patterns that reflect biological data. Each gene was then evolved down its subtree under a GTR+Gamma+Invariable process (i.e., General Time Reversible process, with rates for sites drawn from a gamma plus invariable distribution [20]).

#### Step 3: Dataset production

This step was also performed in a novel way, producing datasets for both combined analysis and source tree estimation that reflect the practice of systematists. We produced datasets of genes appropriate for estimating trees on specific clades (rooted subtrees) within the tree, and also datasets of genes appropriate for estimating the scaffold tree. For each clade dataset, we selected three genes (each evolving on the same tree, but almost always under a different set of parameters); each scaffold dataset was based on 1-4 genes (again, each evolving on the same tree, but often under a different set of parameters). Thus, each clade-based and scaffold dataset provided to the phylogeny estimation routine was itself a combined dataset.

#### Step 4: Estimation of source trees and the combined analysis trees

We followed standard practice here, using RAxML [21] for maximum likelihood (ML) and PAUP* [22] for maximum parsimony (MP) to estimate trees. However, we did not use a partitioned analysis within RAxML, thus somewhat hampering the accuracy of the maximum likelihood analyses.

#### Step 5: Estimation of the supertrees

We used both standard and weighted MRP.

#### Step 6: Performance evaluation

We mostly followed standard practice here, evaluating topological accuracy (with respect to false negative and false positive rates) and running time. We also explored the impact of dataset parameters on topological accuracy.

### Step 1: Generate model trees

For trees having 100, 500 and 1000 taxa, we generated random model trees [23], with 30 replicates generated for the 100 and 500 taxon cases, and ten for the 1000 taxon case. The smaller number of replicates for 1000 taxa was due to the very long run times for the 1000 taxon analyses. We produced the model tree topologies using r8s [24], generating non-ultrametric trees in two steps. First, we generated ultrametric model trees under a Yule pure-birth process with the targeted numbers of taxa and tree height of 1.0. We then modified branch lengths to deviate from ultrametricity by applying a random scaling factor to each edge length in the tree. At the root, the scaling factor was set to 1.0 and progressively altered-parent branch to daughter branches-by adding a value, drawn from a normal distribution with mean zero and standard deviation 0.05. The scaling factors were constrained to be at least 0.05 and no greater than eight. (See appendix for commands used [Additional file 1]).

### Step 2: Evolve gene sequences

For each model tree, we generated a large suite of genes for use in inferring the source trees. Genes for inferring scaffold trees always appeared at the root of the tree and did not go extinct; these are termed universal genes. Five universal genes were evolved for each model tree. The genes we created for the clade-based source trees did not occupy the entire tree and are called non-universal genes.

We simulated 100 non-universal genes for each model tree as follows. We selected a single birth node for each gene by randomly selecting the gene's birth point using the model tree topology and branch lengths. For each gene we evolved a single binary site starting at the root. Initially, the site took state zero, representing the absence of the gene. Branch lengths were normalized so that the distribution was independent of tree height. A decay process *e*^-λ*b*^, where λ is the normalization factor and *b *is the branch length, was tested along all branches leading away from a parent node, and if the birth occurred then the state of the parent node was set to 1, representing the presence of the gene. This node was the "birth" place of the given gene. If the birth did not occur, a recursive call was made to all children. If several children of the same node were returned, then we picked the earliest. If several children of the same node are returned with equal dates, one was selected at random with equal probability.

To determine the lineages for which the given gene was lost, we continued to evolve the binary site starting at the birth node using the same decay process described above. We assigned the loss to the node below the branch on which the loss event occurred, and we allowed multiple loss events. Thus, the process for determining birth and extinction points of the genes produced a connected set of nodes in the tree containing the gene. This subtree constituted the model tree topology for the given gene.

Following generation of the birth-death patterns for the genes, gene sequences, each of length 500, were evolved under the GTR+Gamma+I model, where the parameters of the model were chosen with equal probability from a pool of parameter sets estimated by Ganesh Ganapathy [25] on three biological datasets (Table 2): (a) the Angiosperm data set - 288 aligned DNA sequences of a group of Angiosperms, each of length 4811 [17,26]; (b) the Nematode data set - 682 aligned small subunit rRNA sequences, consisting of 678 species of Nematodes and four outgroups, each of length 1808 [27]; and (c) the rbcL data set - 500 aligned rbcL DNA sequences each of length 1398 [28]. Genes were evolved at a fast, medium or slow rate, implemented by rescaling the model tree branch lengths by a factor of 2.0, 1.0, or 0.1, respectively.

**Table 2:** Gene sequence parameters

Data Set	Substitution Matrix			Base Frequencies.	Prop. Invar. Sites	Gamma
Angiosperm	1.54755	3.67531	1.86115	A = 0.223269	0.2	0.5
		0.93047	4.53303	C = 0.206748		
			1.0	G = 0.256568		
				T = 0.313414		

Nematode	1.24284	3.47484	0.48667	A = 0.300414	0.273196	0.362026
		1.07118	4.38510	C = 0.191363		
			1.0	G = 0.196748		
				T = 0.311475		

rbcL	1.09397	3.12811	0.35141	A = 0.320128	0.101878	0.397524
		1.55972	3.64704	C = 0.176726		
			1.0	G = 0.167462		
				T = 0.335683		

Model parameters estimated on three biological data sets and used for generating DNA sequences.

Universal genes were always slow - reflecting the practice in systematics of using slower evolving genes for higher taxonomic groups. Twenty-five of the non-universal genes were fast, 50 were medium, and 25 slow. We evolved each gene independently down its model tree using the program Seq-Gen [29] (see appendix for the Seq-Gen commands [Additional file 1]).

### Step 3: Data set production

For each model tree, we created DNA sequence data sets for phylogenetic analyses. Data sets differed in the taxa and genes used and whether they were scaffold or clade-based in order to mimic taxon-sampling strategies used by systematists. The result of this process was a collection of data matrices, which we then used for the combined and supertree analyses.

For each clade-based data set, we selected a clade of interest using the same process to select an edge as that used in the birth node selection process above. The child node of the selected edge was returned as the root node of the clade of interest. Clade selection was restricted by setting bounds on the number of extant taxa in a clade to avoid selection of either very small or very large clades. For each 100-taxon model tree, we selected five clades with a clade size of at least 20. For each 500-taxon model tree, we selected 15 clades with a clade size of at least 30, and for each 1000-taxon model tree, we selected 25 clades ranging in size between 30 and 500. We never created more than one clade-based source tree for any clade in the model tree.

For each clade chosen, we selected the three non-universal genes that covered the largest number of taxa in the clade, breaking ties randomly. Once the three non-universal genes were chosen, we restricted the taxa in the clade to only those that had all three of the genes. This process produced clade-based datasets without any missing sequence data, but that may not have contained all the taxa of the specified clade. Since this process could produce datasets with small numbers of taxa, we excluded any clade-based data set that had fewer than ten taxa.

For the scaffold data sets, we used the same technique as in Bininda-Emonds and Sanderson [5], and selected a subset of taxa uniformly at random from the model tree, with a fixed probability *p*, which we called the "scaffold-factor." By design, the scaffold datasets generated by this process had on average *p *× *n *taxa, where *n *is the number of taxa in the model tree. We generated scaffold data sets with a scaffold factor of either 0.20, 0.5, 0.75 or 1.0, for either one, two or four universal genes. The larger scaffold factors were chosen to ensure some model conditions had the taxonomic overlap necessary to potentially reconstruct an accurate supertree. For the smaller scaffold factors, we produced a handful of datasets with such low taxon overlap that it would have been inappropriate to apply either a supertree or a supermatrix analysis (see [[30], pg. 257] for a description of the conditions needed to apply a supertree analysis). These datasets were discarded from our study. Because of the combination of scaffold and clade-based source trees, and because all were larger than some minimum size, we were able to achieve good coverage of most taxa in the model tree.

For each replicate, after all the source tree gene datasets were selected, a "combined dataset" was created for the combined analyses, CA-MP and CA-ML. If a gene happened to be used in more than one source tree dataset, we superimposed the alignments on the different taxon sets to produce a single alignment on the union of these source tree taxon datasets. For example, if a gene were used for source trees on taxon sets *A *and *B *(note these alignments will be identical on *A *∩ *B*), we merged these two alignments into a single alignment on the set *A *∪ *B*. Since the clade-based datasets used only non-universal genes and the scaffold datasets contained only universal genes, this process of merging alignments coming from the same gene only happened for the genes used in clade-based datasets.

### Step 4: Estimation of source trees and the combined analysis tree

For each data matrix, we inferred phylogenies using both MP and ML methods. The MP source trees for the MRP analyses were estimated using the parsimony ratchet implemented in PAUP* [22]. Starting trees were generated from a random addition sequence, and followed by one round of TBR swapping. Once a local optimum was reached, we performed a ratchet iteration. The first step of the ratchet iteration randomly reweighted 25% of the characters with weight 2.0, while keeping the weight of the other characters 1.0. A round of TBR hill-climbing was then performed on the reweighted data matrix. During the second step, the weights on all characters were returned to 1.0, and another round of TBR hill-climbing was performed. (See appendix for commands used.) We performed 50 iterations on the scaffold data sets and 100 iterations on the clade-based data sets, keeping the most parsimonious solutions obtained over all the iterations. Finally, we returned the strict consensus of the most parsimonious trees found.

The MP source trees for the weighted MRP analyses were estimated using a fast MP command (bootstrap nreps = 1000 search=faststep) in PAUP* to analyze 1000 bootstrap replicates. (See appendix for command used.) We chose this approach for generating the wMRP source trees because the parsimony ratchet we used for the MRP input trees was too slow in this context (given the number of bootstrap replicates we analyzed). We returned the majority consensus of these analyses.

To generate the ML input trees for the (unweighted) MRP analysis, we used RAxML [21] in its default setting (GTRMIX), returning the single best tree. We estimated the ML source trees for weighted MRP using the fast bootstrap version of RAxML, analyzing 100 bootstrap replicates in its GTRMIX setting. (See appendix for commands used.) The smaller number of bootstrap replicates for the ML analyses, as compared to the MP analyses, was necessary due to longer runtimes per replicate. Also, the weighted MRP-ML analyses could only be performed on the 100 taxon datasets due to very long runtimes on the 500 and 1000 taxon cases.

For the combined analyses, phylogenies were estimated on the supermatrix created from the source-tree matrices using both maximum parsimony (CA-MP) and maximum likelihood (CA-ML). MP analyses consisted of five iterations of the parsimony ratchet, using the same implementation described for the input tree reconstructions, returning the majority consensus of the most parsimonious trees found. ML analyses used RAxML in its GTRMIX default setting for the 100- and 500-taxon data sets and in its GTRCAT default setting for the 1000-taxon data sets. GTRCAT was used for the largest data sets due to running time and memory constraints. GTRMIX uses the same search algorithm as GTRCAT, but recomputes the likelihood score on the final tree. Thus, both variants of RAxML methods produce trees with the same topology, differing only in the numeric parameters.

### Step 5: Estimation of the supertrees

For the supertree analyses, we used the MP and ML source trees as input for the MRP method, and the bootstrap MP and bootstrap ML source trees as input to the weighted MRP method (wMRP); thus, MRP-MP and wMRP-MP had slightly different source trees, and MRP-ML and wMRP-ML also had slightly different source trees. For the wMRP analyses, the bootstrap proportions from the input tree analyses were used as the branch lengths. Thus we had a total of four different supertree reconstructions (MRP-MP, MRP-ML, wMRP-MP, and wMRP-ML). The MRP method was used because it is the most popular of the various supertree methods, and wMRP was used because Bininda-Emonds and Sanderson [5] results indicated that wMRP-MP performed better than MRP-MP and combined analyses. We used r8s [24] to produce the matrices for the MRP and wMRP analyses from the given source trees. For the MRP analyses, we analyzed the supertree matrices using 50 iterations of the parsimony ratchet described above. Since the parsimony ratchet in PAUP* will not accept weights for branches, in order to perform the wMRP analyses we used a weighted parsimony search (with 100 random sequence additions, TBR branch swapping, and maxtrees = 1000). (See appendix for command used.) For both MRP and wMRP analyses, we returned the majority consensus of the most parsimonious supertrees returned by the search.

### Step 6: Performance evaluation

Steps 1 through 5 produced results for four supertree methods and two combined analysis methods. The supertree methods were MRP based upon MP trees (MRP-MP), MRP based upon ML trees (MRP-ML), weighted MRP based upon MP trees (wMRP-MP), and weighted MRP based upon ML trees (wMRP-ML)), and the combined analysis methods were based upon either MP (CA-MP) or upon ML (CA-ML). We calculated topological error using the false negative (FN) rate-the number of edges present in the model tree but not in the estimated tree, divided by the number of internal edges in the model tree (*n *- 3 where *n *is the number of taxa)-and the false positive (FP) rate-the number of edges present in the estimated tree but not in the model tree, divided by the number of internal edges in the estimated tree. We also calculated the arithmetic mean of the FN and FP rates, which we refer to as the "average topological error". Note that when the trees being compared are binary, the average topological error is equivalent to the normalized Robinson-Foulds (RF) distance [31]. For each model condition, we calculated the average error rates and standard errors.

We recorded the running time of each method on each dataset. Because the analyses were run under Condor (a distributed software environment [32]), the running times (for the larger datasets, especially) are inexact, and larger than they would be if run on a dedicated processor. Running times are provided to give an approximate estimation of the time needed to perform these analysis. We report the maximum and minimum running time for each model condition.

Finally, we explored the impact of (1) the topological error of the source trees, (2) the scaffold factor, (3) the number of scaffold genes, and (4) the number of taxa on the topological error of the resultant supertrees.

## Results and Discussion

### Relative performance of methods

**Figure 1:**
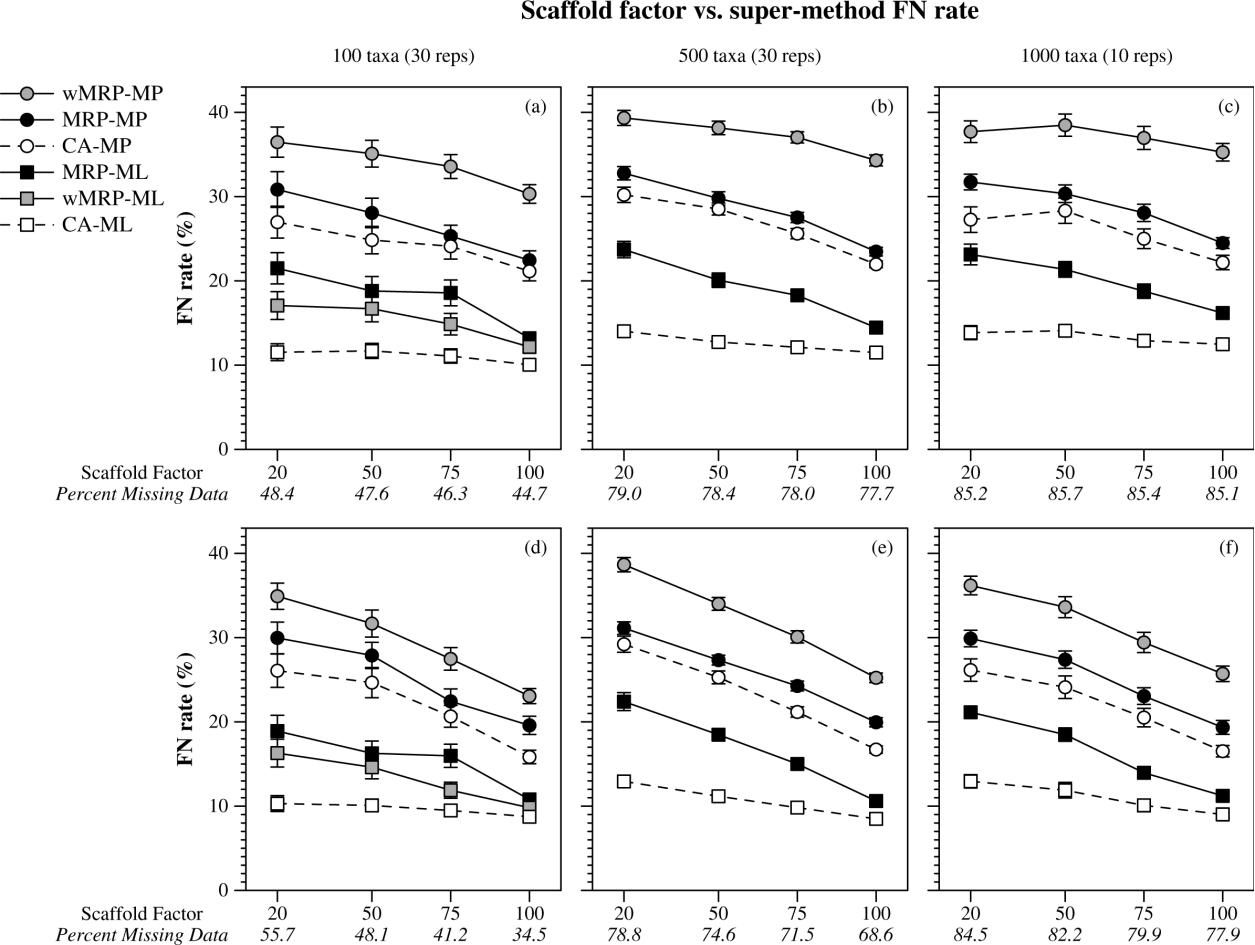
**Scaffold factor vs. super-method FN rate**. False Negative (FN) rates (means with standard error bars) for supertree and supermatrix reconstructions as a function of the scaffold factor. Values in italics on the x-axis are the average percent of missing data in the data matrices of the combined data sets for that scaffold factor. Graphs a-c are for data sets with one scaffold gene, and d-f for four scaffold genes.

**Figure 2:**
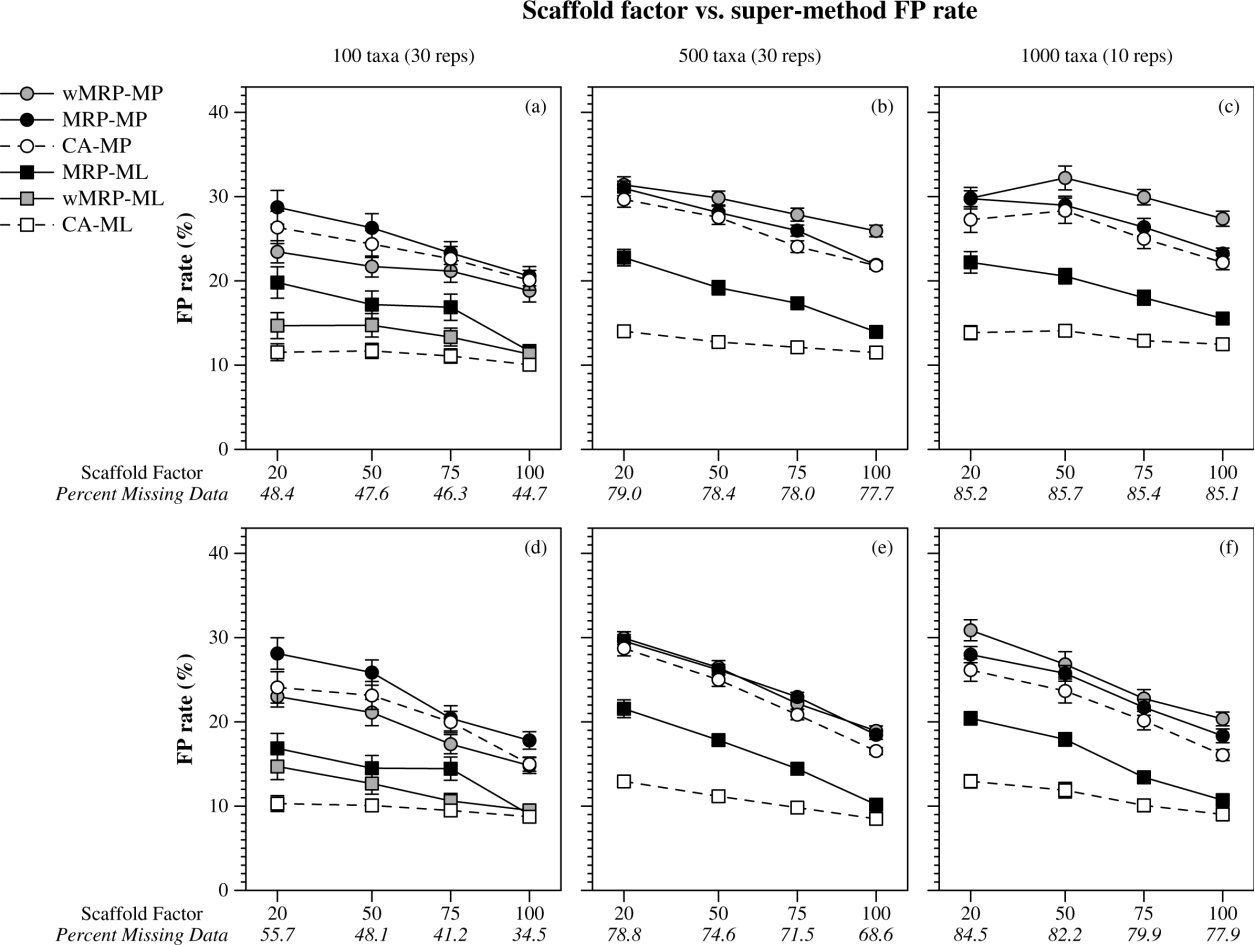
**Scaffold factor vs. super-method FP rate**. False Positive (FP) rates (means with standard error bars) for supertree and supermatrix reconstructions as a function of the scaffold factor. Values in italics on the x-axis are the average percent of missing data in the data matrices of the combined data sets for that scaffold factor. Graphs a-c are for data sets with one scaffold gene, and d-f for four scaffold genes.

**Figure 3:**
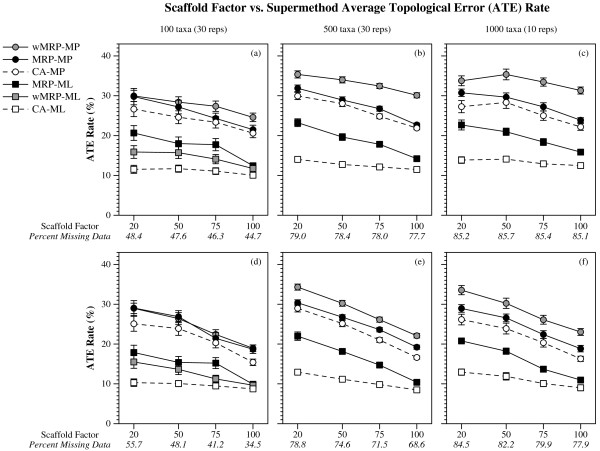
**Scaffold factor vs. super-method avg. topological error**. Average topological error (means with standard error bars) for supertree and supermatrix reconstructions as a function of the scaffold factor. Values in italics on the x-axis are the average percent of missing data in the data matrices of the combined data sets for that scaffold factor. Graphs a-c are for data sets with one scaffold gene, and d-f for four scaffold genes.

**Figure 4:**
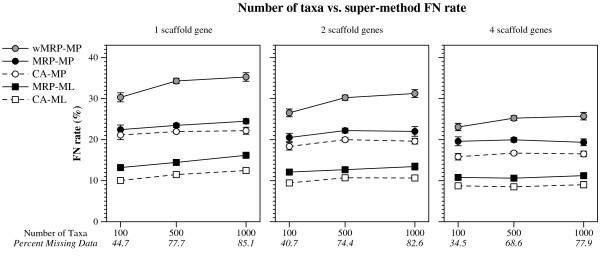
**Number of taxa vs. super-method FN rate**. FN rates (means with standard error bars) for supertree and supermatrix reconstructions as a function of the number of taxa in the model tree and the number of scaffold genes. Values in italics on the x-axis are the average percent of missing data in the data matrices of the combined data sets for that scaffold factor. Only data sets with 100% scaffold factors are presented.

**Figure 5:**
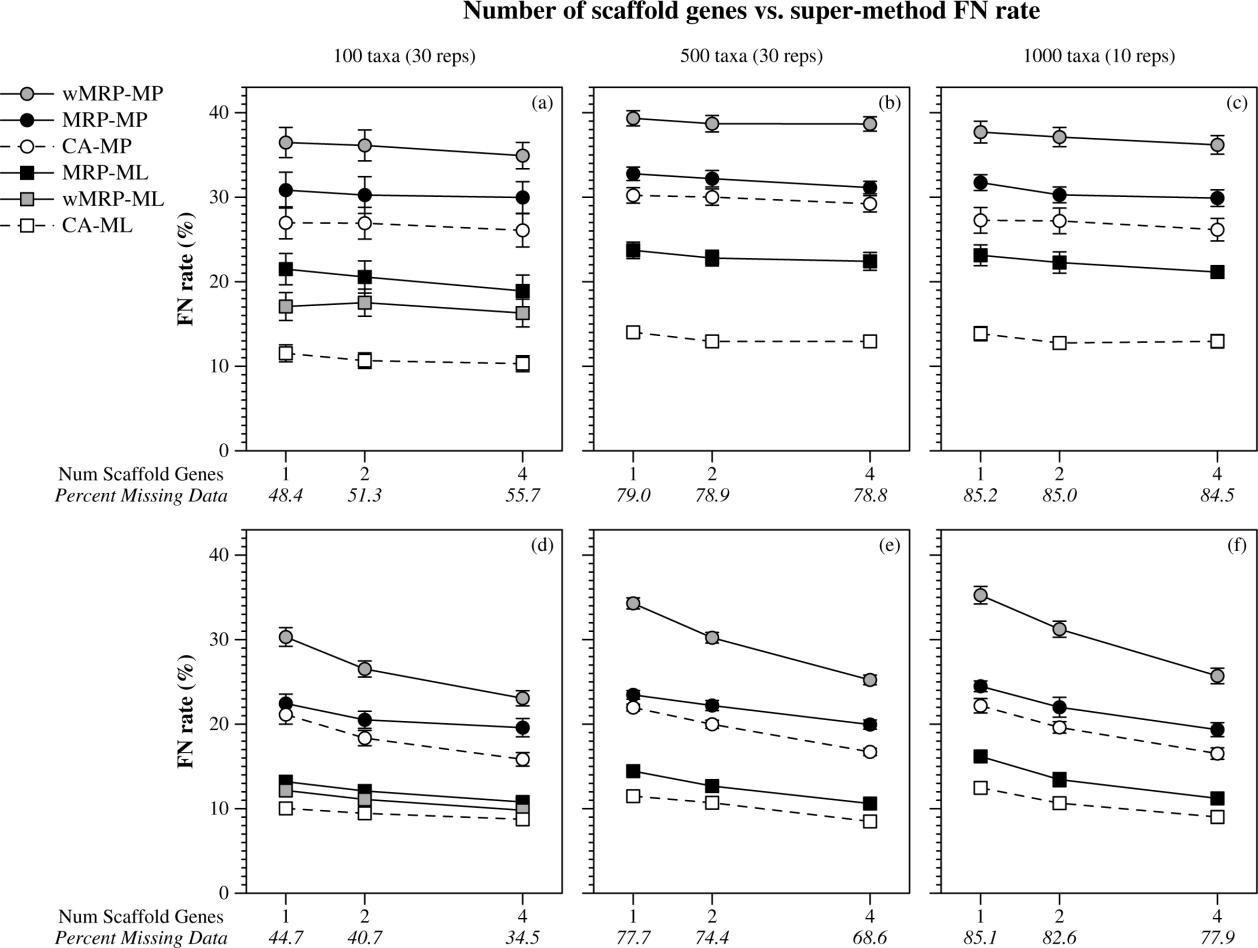
**Number of scaffold genes vs. super-method FN rate**. FN rates (means with standard error bars) for supertree and super-matrix reconstructions as a function of the number of scaffold genes used in the scaffold reconstruction. Values in italics on the x-axis are the average percent of missing data in the data matrices of the combined data sets for that scaffold factor. Graphs a-c and d-f are for data sets with 20% and 100% scaffold factors, respectively.

Interestingly, the six methods we studied had roughly the same relative topological accuracy (measured with respect to FN and FP rates and to average topological error) under most model conditions. CA-ML was consistently the best method, with *much *lower topological error than the other methods for most model conditions. Following CA-ML were the other ML-based methods-wMRP-ML, and MRP-ML, in that order-and then the three MP-based methods-CA-MP, MRP-MP, and wMRP-MP, usually in that order

(Figures 1, 2 and 3). CA-ML's advantage was substantial for cases where the scaffold factor was less than 100% (with the biggest advantage for the smallest scaffold factors), and this advantage increased slightly with the number of taxa and decreased with the number of scaffold genes (Figures 4 and 5).

In comparing the performance of the different algorithms (combined analysis or supertree method, based upon either maximum parsimony or maximum likelihood), we discovered that certain algorithm design choices had a large impact on the topological accuracy of the trees that were constructed. In particular, the choice of optimization problem, i.e. whether we used maximum likelihood or maximum parsimony, had the largest impact on the final accuracy, with methods that used maximum likelihood (CA-ML, wMRP-ML, and MRP-ML) as a group more accurate than the methods based upon maximum parsimony (CA-MP, wMRP-MP, and MRP-MP). The second most significant algorithmic choice was whether we performed a combined or a supertree analysis, with CA-MP more accurate than MRP-MP and wMRP-MP, and similarly CA-ML more accurate than MRP-ML and wMRP-ML.

**Figure 6:**
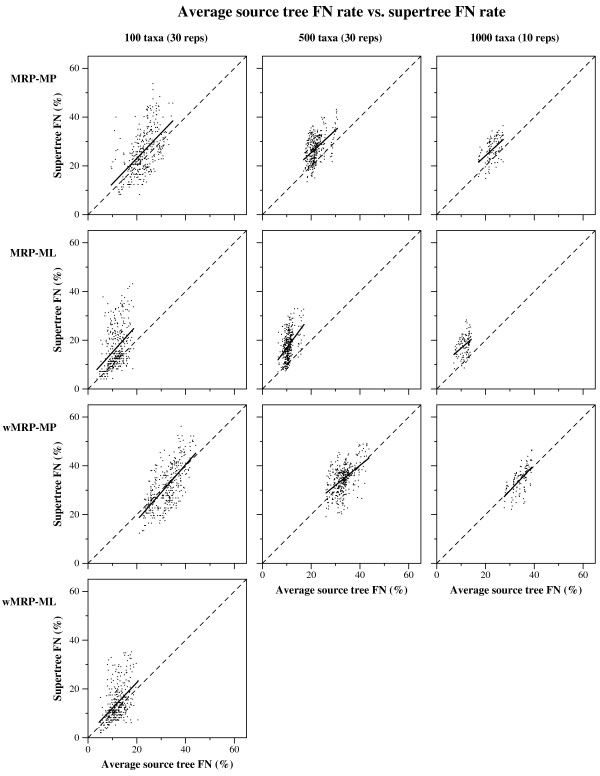
**Average source tree FN rate vs. supertree FN rate**. Average source tree FN rates against supertree FN rates. Each point represents a single replicate for a single model condition. The solid line is a regression line. The dotted line represents supertree constructions that have the same FN as the average source tree given as input. Points above the dotted line correspond to supertrees that are less topologically accurate than the average source tree, while points below the line correspond to supertrees that improved upon the average accuracy of the source trees.

The first of these observations (that ML-based analyses were more accurate than MP-based analyses) is in some ways not surprising. Supertree methods are sensitive to error in their source trees, so improving the accuracy of the source trees will improve the accuracy of the supertrees. Furthermore, our study showed that source trees based upon ML were on average more accurate than source trees based upon MP. ML source trees usually had less than 20% FN error, and MP source trees usually had more than 20% (Figure 6). These two observations suggest that supertrees based upon ML source trees should be more accurate than supertrees based upon MP source trees.

However, while it is not surprising that trees estimated using ML are more accurate than trees estimated using MP, the performance of ML in our study, particularly in the combined analyses, is still noteworthy because our data analyses did not include partitioning of the datasets. All the sequence datasets we generated were obtained by concatenating gene data sets; maximum likelihood analyses of these datasets then proceeded without using the "partitioned analysis" option. Since each gene in the dataset could evolve under a different model of evolution, the concatenated data sets were not guaranteed to evolve under the same GTR model. Therefore, our ML analyses were performed under a simpler (single GTR matrix) model, even though the data were generated under a more complex model. This treatment of the data has the potential to reduce the accuracy of all our ML-estimated trees (whether ML source trees or ML combined analysis trees), in comparison to what they might have been if they had been estimated under a partitioned analysis for each gene. Therefore, *the improved topological accuracy of the ML-based methods in comparison to the MP-based methods shows that maximum likelihood is likely to be more accurate than MP*.

### Running time

Table 3 provides information on the running times of the methods studied here. Since these methods were run under Condor (a distributed system lacking dedicated processors), these numbers should be considered approximate and are given only as an indication of the general trends. Running times for wMRP-ML were prohibitively high for the 500 and 1000 taxon datasets, making it infeasible to use wMRP-ML on datasets of these sizes. Almost as problematic is wMRP-MP, which can be extremely slow on some 1000 taxon datasets. Combined analysis using maximum likelihood takes more time than combined analysis using maximum parsimony or MRP-ML and MRP-MP, but is still acceptable in its computational requirements (although it requires more than a day of analysis on the 1000 taxon datasets).

**Table 3:** Running time

Num. Taxa	Scaffold Factor	Num. Scaff. Genes	MRP				wMRP				Comb. Analysis	
			**MP**		**ML**		**MP**		**ML**		**MP**	**ML**
100	20%	1	0:02:26 (0:00:16)		0:04:12 (0:00:17)		0:01:59 (0:00:30)		7:00:43 (0:07:22)		0:00:19	0:09:22
		2	0:02:46 (0:00:18)		0:04:33 (0:00:17)		0:01:59 (0:00:29)		13:09:51 (0:08:19)		0:00:20	0:14:49
		4	0:02:46 (0:00:18)		0:04:48 (0:00:17)		0:02:02 (0:00:30)		13:12:03 (0:09:27)		0:00:22	0:24:19
	50%	1	0:02:31 (0:00:17)		0:04:52 (0:00:17)		0:02:02 (0:00:34)		13:27:05 (0:07:26)		0:00:20	0:19:28
		2	0:02:52 (0:00:17)		0:04:43 (0:00:17)		0:02:00 (0:00:29)		13:34:02 (0:08:30)		0:00:19	0:18:45
		4	0:02:36 (0:00:19)		0:04:47 (0:00:17)		0:02:03 (0:00:27)		13:29:32 (0:09:39)		0:00:21	0:11:47
	75%	1	0:02:34 (0:00:18)		0:04:40 (0:00:18)		0:02:10 (0:00:38)		13:37:31 (0:09:14)		0:00:20	0:11:22
		2	0:02:37 (0:00:21)		0:05:08 (0:00:19)		0:02:08 (0:00:31)		7:51:22 (0:04:47)		0:00:20	0:14:09
		4	0:02:39 (0:00:19)		0:05:25 (0:00:19)		0:02:12 (0:00:27)		16:08:55 (0:06:31)		0:00:22	0:17:46
	100%	1	0:03:12 (0:00:19)		0:04:49 (0:00:18)		0:02:15 (0:00:40)		16:04:16 (0:00:46)		0:00:19	0:15:59
		2	0:02:42 (0:00:19)		0:05:12 (0:00:18)		0:02:15 (0:00:31)		16:23:16 (0:00:32)		0:00:20	0:15:59
		4	0:02:51 (0:00:19)		0:05:24 (0:00:17)		0:02:27 (0:00:27)		14:12:23 (0:00:30)		0:00:21	0:16:42

500	20%	1	0:38:08 (0:14:00)		0:42:23 (0:10:30)		4:33:43 (4:11:26)				0:31:32	8:18:55
		2	0:36:41 (0:13:12)		0:54:00 (0:14:02)		4:16:10 (3:52:54)				0:30:03	8:11:45
		4	0:36:15 (0:12:38)		0:43:26 (0:10:26)		3:40:18 (3:16:18)				0:31:41	10:46:11
	50%	1	0:35:50 (0:12:57)		0:50:02 (0:11:11)		5:15:58 (4:54:09)				0:36:43	10:28:04
		2	1:04:27 (0:23:12)		0:53:11 (0:12:19)		5:16:34 (4:50:07)				0:33:17	11:11:52
		4	0:46:12 (0:15:36)		0:59:05 (0:13:42)		5:12:08 (4:42:06)				0:31:30	8:40:21
	75%	1	0:38:20 (0:13:45)		0:54:45 (0:12:56)		7:03:10 (6:40:10)				0:37:44	7:02:24
		2	0:37:54 (0:12:30)		0:52:09 (0:11:05)		6:34:58 (6:07:25)				0:34:19	7:53:34
		4	0:41:21 (0:12:39)		0:57:47 (0:11:43)		3:52:45 (3:18:24)				0:29:39	8:38:06
	100%	1	0:44:33 (0:15:07)		0:57:14 (0:11:53)		7:04:20 (6:43:18)				0:35:42	7:51:45
		2	0:44:38 (0:14:23)		1:09:41 (0:14:36)		12:33:24 (11:56:14)				0:37:26	7:59:34
		4	0:43:44 (0:12:02)		1:05:59 (0:11:38)		5:33:52 (4:55:00)				0:26:27	7:10:35

1000	20%	1	3:27:39 (2:21:39)		3:14:56 (1:47:23)		85:59:18 (84:53:20)				6:09:44	30:51:13
		2	3:22:14 (2:18:39)		3:26:56 (1:47:54)		30:34:14 (29:53:19)				5:46:56	28:05:57
		4	4:23:55 (3:05:25)		3:21:37 (1:51:27)		83:56:34 (82:42:34)				6:15:50	27:58:00
	50%	1	3:22:33 (2:18:18)		4:22:00 (2:18:23)		75:21:00 (74:11:14)				7:36:14	33:21:22
		2	3:36:43 (2:24:07)		3:28:32 (1:37:26)		54:19:41 (53:30:45)				6:14:55	29:23:03
		4	3:15:25 (2:01:12)		4:06:33 (2:00:27)		41:23:37 (40:19:38)				11:13:18	29:28:29
	75%	1	4:12:25 (2:38:59)		4:18:28 (1:58:46)		88:13:11 (87:10:11)				8:07:53	33:39:42
		2	4:01:05 (2:24:18)		4:22:16 (2:00:57)		67:17:02 (66:12:47)				6:16:32	28:41:11
		4	4:11:41 (2:22:43)		4:34:12 (1:40:56)		50:49:52 (49:25:52)				5:33:09	29:08:11
	100%	1	5:29:37 (3:26:03)		4:29:46 (1:52:53)		174:47:23 (173:41:33)				7:56:46	34:14:48
		2	4:26:05 (2:39:56)		5:53:10 (2:30:23)		295:10:35 (293:51:27)				6:02:16	27:11:45
		4	4:54:31 (2:43:09)		4:43:11 (1:51:43)		174:52:35 (172:49:10)				4:49:07	24:33:39

Average running time for each of the six methods. For the four supertree methods, the time to compute just the supertree is given in parentheses following the full running time, which includes the time taken to compute source trees. Running times are given in hours:minutes:seconds.

### Comparison with earlier studies

The most appropriate studies for comparison with the work presented here are those by Bininda-Emonds and Sanderson [5] and Criscuolo et al. [13] since they both performed simulation studies using MRP and combined analysis approaches. However, Bininda-Emonds and Sanderson only examined CA-MP, wMRP-MP, and MRP-MP, while Criscuolo et al. only examined CA-ML and MRP-ML; our study is, thus the first to consider wMRP-ML as a supertree method as well as the first to compare MP-based "super-methods" to ML-based super-methods. Direct, quantitative comparisons with their studies are somewhat complicated because they used different metrics for assessing the topological accuracy of estimated trees relative to the true trees: Criscuolo et al. used quartet distances and Bininda-Emonds and Sanderson used the consensus fork index (CFI). We, therefore, restrict our comparisons to qualitative differences.

The result common to all three studies is that for each fixed optimality criterion (MP or ML), combined analysis is more accurate than MRP. However, the studies differ in terms of the magnitude of the improvement obtained by combined analysis over MRP, with our study finding much larger differences (especially for the small scaffold factors). Beyond the relative performance of MRP and combined analysis, the three studies come to different conclusions. Bininda-Emonds and Sanderson found that wMRP-MP was more accurate than CA-MP, whereas, across all model conditions, our study found that CA-MP was more accurate than wMRP-MP, having much lower FN error rates and comparable FP error rates. We also found that wMRP-ML was not as accurate as CA-ML for 100 taxon datasets. Since neither earlier study evaluated wMRP-ML as a supertree method, our findings with respect to its performance cannot be compared to their findings, except insofar as our findings are helpful in understanding wMRP as a generic supertree method.

The differences between our findings and those of Bininda-Emonds and Sanderson and Criscuolo et al. could be due to several factors. Since our results show that increasing the number of taxa increases the relative advantage of combined analysis over MRP and wMRP, we suspect that one factor is likely the number of taxa in the experiments: we examined datasets with between 100 taxa and 1000 taxa, while Bininda-Emonds and Sanderson explored datasets with at most 32 taxa and Criscuolo et al. explored 48 and 96 taxon datasets. Since most recent empirical supertree studies have included upwards of 200 taxa (see Table 1), and it is likely that future empirical supertree analyses will also tend to be in the range of our analyses, our results may be a better indicator of the relative performance of MRP supertree and combined analysis methods (and in particular, of wMRP-MP and CA-MP) for current uses of these methods. A second factor could be our simulation methodology. One of the main differences between the simulation methodology we used and those used by others is the taxon sampling procedure: our datasets included clade-based source trees and scaffold source trees, while the technique used by both Bininda-Emonds and Sanderson and Criscuolo et al. produced only scaffold datasets, because their taxa were always randomly selected from the full dataset. (Bininda-Emonds and Sanderson used 25%, 50%, 75%, and 100%, and Criscuolo et al. used 25% and 75% scaffold factors.) While the comprehensiveness of taxon sampling for in-groups in biological studies varies depending on the purpose of the study, the resources available to the researchers, and the ability to collect or access source material, there is almost always a clear non-random distribution of taxon-sampling effort in most of the individual trees that would be used as input for a supertree method or for producing a supermatrix. Thus, it is likely our technique better replicates systematic practice than theirs. To test this expectation, we designed an additional experiment to see if using only scaffold-based source trees would result in findings more similar to those produced by Bininda-Emonds and Sanderson and Criscuolo et al.

Using our 100-taxon model trees, and using the same methodology described in the *Methods *section, we generated a collection of "all-scaffold" datasets for analysis by MRP-ML and CA-ML. For each model condition, we generated 30 replicate datasets. First, we generated 100 universal genes (50 slow and 50 medium), under different GTR+Gamma+I models. For the MRP-ML analyses, we produced six source trees, each based upon four genes, and sampling taxa at random for the specified scaffold factor. Note that different source trees could use genes used by other source trees, but within a source tree all four genes were different. These gene matrices were combined into a single matrix for the combined analysis. We then analyzed the datasets using MRP-ML and CA-ML, and scored each tree for its false negative and false positive rate. We also recorded the topological error in the estimated source trees.

**Figure 7:**
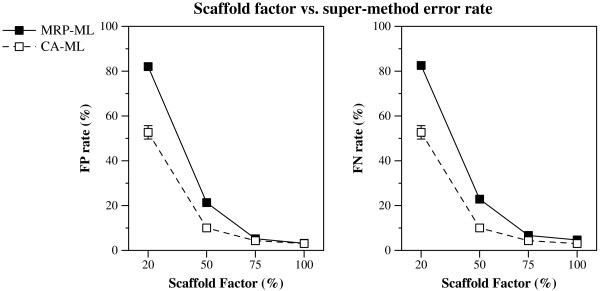
**All-scaffold data scaffold factor vs. super-method FN rate**. FN and FP rates (means with standard error bars) for supertree and supermatrix reconstructions as a function of the scaffold factor, for datasets where all source tree datasets are scaffold datasets containing four universal genes.

**Figure 8:**
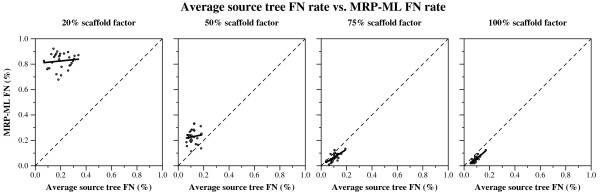
**All-scaffold data average source tree FN rate vs. supertree FN rate**. Average source tree FN rates against supertree FN rates. Each point represents a single replicate for a single model condition with a 100-taxon model tree and datasets for which all source trees are estimated using scaffold datasets containing four universal genes. The solid line is a a regression line. The dotted line represents supertree constructions that have the same FN as the average source tree given as input. Points above the dotted line correspond to supertrees that are less topologically accurate than the average source tree, while points below the line correspond to supertrees that improved upon the average accuracy of the source trees.

The patterns we saw for these "all-scaffold" model conditions were mostly consistent with our first experiment, but also provide interesting contrasts (contrast Figure 7 to Figures 1 and 2). The two experiments show roughly the same relative performance between CA-ML and MRP-ML: for the lowest scaffold factors (20% and 50%) CA-ML is much more accurate than MRP-ML, while for the largest scaffold factors (75% and 100%) they have almost identical performance. As before, we see that scaffold factor impacts the accuracy of both methods, but in this experiment the impact is greater: at the lowest scaffold factors (20% and 50%) both methods produce very inaccurate trees (with MRP-ML clearly worse), and at the highest scaffold factors they produce highly accurate trees. Since the scaffold factor is a pretty close approximation to the amount of nucleotide data in the matrix (i.e., for scaffold factor of 20%, the alignment will have about 20% nucleotides and 80% missing data), this suggests that the amount of missing data for these all-scaffold datasets has a large impact on the accuracy of both supertree and combined analysis methods. Note, however, that CA-ML seems somewhat more robust to missing data than MRP-ML. Given MRP-ML's particularly poor accuracy on the sparse scaffold datasets, we examined the error rates in the source trees to see if the problem was due to poor source trees (Figure 8). Interestingly, the ML source trees had only moderately high error rates (about 20%) for the lowest scaffold rate-a case where MRP-ML had average error above 80%; a similar, but less extreme, situation presents for the 50% all-scaffold datasets. We provide the following possible explanation for these results: when the datasets are all very sparse scaffold datasets, there may not be enough overlap in the source trees to provide enough phylogenetic signal, thus hampering potentially any supertree method. While supermatrix methods are also impacted negatively, they are somewhat more robust to missing data.

At the other end of the spectrum, when all the source trees are dense scaffold datasets, we see that both MRP-ML and CA-ML produce highly accurate trees, even improving in accuracy over the source trees (Figure 8). This interesting phenomenon also has a potential explanation. First, different source trees can be helpful for different parts of the tree, and so may provide complementary information about the tree; this allows supertree methods (as well as supermatrix methods) to return a more accurate tree on the full set of taxa than the average source tree.

A comparison between the results we see for all-scaffold datasets and the combined types of source tree datasets (some scaffold and some clade-based) shows that however one estimates trees (supertree or supermatrix), all-scaffold datasets tend to result in very poor trees *except *when they are all very dense. We also saw that the mixed source tree dataset conditions had the best accuracy when some source trees are dense, but that reasonably good results could be obtained from only moderately dense scaffold datasets *if *analyzed using CA-ML. Furthermore, MRP seems more sensitive to the problem of sparse taxonomic sampling than combined analysis, further lending support to the hypothesis that MRP is generally inferior to combined analysis.

## Conclusions

Our study has two main contributions. First, we provide a new experimental methodology, SMIDGen (available from the first author), for generating simulated multi-marker datasets, and we show that SMIDGen can be used to evaluate supertree and combined analysis methods under a range of conditions that reflect both biological processes and systematic practice. The datasets and model trees used in this study are available as benchmarks in our online supplementary material, at http://www.cs.utexas.edu/users/mswenson/pubs/. Second, we show that combined analysis using maximum likelihood produces more accurate trees than all the other methods we tested, with the degree of improvement increasing with the number of taxa and decreasing with the density of the scaffold tree. Third, we show that taxonomic sampling strategies for the multi-marker datasets affects all phylogenetic analyses, but that MRP-ML is particularly impacted when all the markers are sparsely sampled from the full set of taxa. These results together provide evidence that combined analysis using maximum likelihood (CA-ML) should be used instead of MRP when possible. These results also suggest that the selection of markers for large-scale multi-marker phylogenetic analyses should be done with care, ensuring that a sufficiently large number of markers provide dense coverage within clades, and using sparsely sampled datasets (perhaps) only as needed. However, further research is needed to determine the impact of adding sparsely sampled datasets to an otherwise "good" multi-marker dataset.

Our recommendation to use combined analysis to assemble trees from maximum likelihood source trees thus argues for a computationally intensive approach to large-scale phylogenetics. However, with the availability of fast and highly accurate software for maximum likelihood (e.g., RAxML and GARLI [33]), such combined analyses should not pose a substantial computational problem. Finally, our conclusions are limited specifically to a comparison of MRP and combined analysis, as we did not test any other supertree methods. This limitation was due to the fact that most other supertree methods require the input source trees to be rooted, and our simulation process does not make it easy to locate roots within the estimated source trees. Thus, we leave open the possibility that some of these other supertree methods may outperform combined analysis using ML.

## Competing interests

The authors declare that they have no competing interests.

## Authors' contributions

MSS designed SMIDGen, designed and performed the simulation study, and drafted the manuscript. FB advised MSS on the software design of SMIDGen and helped draft the manuscript. MSS and FB jointly implemented SMIDGen. TW conceived the study, assisted in the design and analysis of the simulation study, and helped draft the manuscript. CRL led the design of SMIDGen, assisted in the design and analysis of the simulation study, and revised the manuscript. All authors read and approved the final manuscript.

## Supplementary Material

Additional file 1 **Appendix**. The appendix includes the commands used to perform the simulation study. Click here for file
