# Supplementary material for: A simulation study comparing supertree and combined analysis methods using SMIDGen
Source: Algorithms Mol Biol. 2010 Jan 4;5:8. doi: 10.1186/1748-7188-5-8 (PMC2837663; doi:10.1186/1748-7188-5-8)
Supplement: Additional file 1 — Appendix. The appendix includes the commands used to perform the simulation study. [file 1748-7188-5-8-S1.PDF]

# Appendix for SMIDGen Supertree Simulation Study

M. Shel Swenson*[1], François Barbançon[2], Tandy Warnow[1], C. Randal Linder[3]

[1]Department of Computer Sciences, The University of Texas at Austin, Austin TX, USA
[2]Microsoft, Redmond WA, USA
[3]Section of Integrative Biology, The University of Texas at Austin, Austin TX , USA

Email: M. Shel Swenson*- mswenson@cs.utexas.edu;

*Corresponding author

## 1 Details of Commands Used in Data Generation

### 1.1 r8s command

The command given to r8s, to construct initial model trees is as follows:

*r8s command.*– `simulate diversemodel=yule_c T=1 ntaxa=`*desired_number_of_taxa* `nreps=1 seed=`*random_seed* `speciation=1 charevol=yes ratemodel=normal startrate=1 changerate=0.05 infinite=yes minrate=0.05 maxrate=8;`

### 1.2 Seq-Gen commands

The commands given to Seq-Gen for each of the three model conditions in Table 2 in the main text (named after the biological dataset on which they are based) are as follows:

**Angiosperm data set:** `-on -z 500 -mGTR -a 0.5 -i 0.2`
`-r 1.54755 3.67531 1.86115 0.93047 4.53303 1.0`
`-f 0.223269 0.206748 0.256568 0.313414 -s` *mean*

**Nematode data set:** `-on -z 500 -mGTR -a 0.362026 -i 0.273196`
`-r 1.24284 3.47484 0.48667 1.07118 4.38510 1.0`
`-f 0.300414 0.191363 0.196748 0.311475 -s` *mean*

**rbcL data set:** `-on -z 500 -mGTR -a 0.397524 -i 0.101878`
`-r 1.09397 3.12811 0.35141 1.55972 3.64704 1.0`
`-f 0.320128 0.176726 0.167462 0.335683 -s` *mean*

### 1.3 Tree Inference Methods:

**Parsimony ratchet comands.** The PAUP* block used to perform the parsimony ratchet search is as follows:

```
begin paup;
set autoclose=yes warntree=no warnreset=no
notifybeep=no monitor=yes taxlabels=full;
log file=logfile replace;
set criterion=parsimony;
pset collapse=no;
                    [!][!*** Replicate 0 (initial tree) ***]
```

```
   hsearch addseq=random nreps=1 rseed=<random_seed>
swap=TBR multrees=no dstatus=60;
savetrees file=<tree_file> format=altnex replace;
   Then, for each integer i from 1 to n, [!][!*** Replicate i ***]
   weights list of character weights (chosen as described in Methods section);
hsearch start=current swap=TBR multrees=no dstatus=60;
weights 1:all;
hsearch start=current swap=TBR multrees=no dstatus=60;
savetrees file=<tree_file> format=nexus append;
                    [!][!*** Determining consensus trees ***]
   set MaxTrees=<number_of_replicates>
gettrees file=<tree_file> allblocks=yes warntree=no;
contree all / strict=yes majrule=yes
treefile=contreefile replace;
tcondense collapse=no deldupes=yes;
savetrees file=treefile replace=yes format=altnex;
log stop; end; quit warntsave=no;
```

**Maximum parsimony bootstrap commands.** The PAUP* commands used in MP bootstrap analysis are as follows:

```
begin paup;
set criterion=parsimony maxtrees=1000 increase=no
storetreewts=yes;
bootstrap bseed=<random_seed> nreps=1000 search=faststep
treefile=<tree_output_file> replace=yes;
savetrees file=<consensus_output_file> replace=yes
savebootp=brlens from=1 to=1 format=altnex;
gettrees file=<tree_output_file> storetreewts=yes mode=3;
savetrees file=<output_file> replace=yes format=phylip;
contree /majrule=yes strict=no usetreewts=yes
treefile=<consensus_output_file> replace;
quit; end;
```

**Maximum likelihood commands.** RAxML commands used in ML analyses are as follows:

Command for ML source trees and 100- and 500-taxon CA-ML analysis:

```
raxmlHPC -s <phylip_alignment_file> -n <output_suffix> -m GTRMIX
-w <working_directory>
```

Command for 1000-taxon CA-ML analysis:

```
raxmlHPC -s <phylip_alignment_file> -n <output_suffix> -m GTRCAT
-w <working_directory>
```

**Maximum likelihood boostrap commands.** RAxML commands used in ML bootstrap analysis are as follows:

```
raxmlHPC -s <phylip_alignment_file> -n <output_suffix> -m GTRMIX
-w <working_directory>
```

(computes a ML tree which we will annotate with bootstrap values)

```
raxmlHPC -s <phylip_alignment_file> -n <bootstrap_analysis_output_suffix>
-m GTRMIX -# 100 -b <random_seed> -w <working_direcory>
```

(computes ML trees from 100 bootstrap replicates)

```
raxmlHPC -f b -s <phylip_alignment_file> -n <bootstrap_tree_output_suffix>
-m GTRMIX -z <boostrap_output_file> -t <ml_tree_file>
-w <working_directory>
```

(annotates the tree computed in the first step with bootstrap values)

**Weighted maximum parsimony search commands.** The PAUP* commands used in weighted MP analysis are as follows:

```
begin paup;
set criterion=parsimony maxtrees=1000 increase=no;
hsearch start=stepwise addseq=random nreps=100 swap=tbr;
lter best=yes;
savetrees file=<output_file> replace=yes format=altnex;
contree all/ strict=yes majrule=yes treefile=<consensus_output_file> replace=yes;
quit; end;
```
